# Acoustic over-exposure triggers burst firing in dorsal cochlear nucleus fusiform cells

**DOI:** 10.1016/j.heares.2011.10.008

**Published:** 2012-01

**Authors:** Nadia Pilati, Charles Large, Ian D. Forsythe, Martine Hamann

**Affiliations:** a Department of Cell Physiology and Pharmacology, Maurice Shock Medical Science Building, University of Leicester, University Road, Leicester LE1 9HN, UK; b Neuroscience CEDD, GlaxoSmithKline S.p.A., Via Fleming 4, Verona 37135, Italy; c MRC Toxicology Unit, Hodgkin Building 1, University of Leicester, UK

**Keywords:** ABR, auditory brainstem response, ACSF, artificial cerebrospinal fluid, AP, action potential, AOE, acoustic over-exposure, CNQX, 6-cyano-7-nitroquinoxaline-2,3-dione, CV, coefficient of variation, Cw, cartwheel cells, DCN, dorsal cochlear nucleus, DL-AP5, DL-2-amino-5-phosphonopentanoic acid, DNQX, 6,7-dinitroquinoxaline-2,3-dione, FCs, fusiform cells, ½ *F*_max_, half maximal frequency, HVA, high voltage activated, ISI, inter-spike intervals, *F*_max_, maximal frequency, N.S., non significant, SPL, sound pressure level, *V*_m_, membrane potential

## Abstract

Acoustic over-exposure (AOE) triggers deafness in animals and humans and provokes auditory nerve degeneration. Weeks after exposure there is an increase in the cellular excitability within the dorsal cochlear nucleus (DCN) and this is considered as a possible neural correlate of tinnitus. The origin of this DCN hyperactivity phenomenon is still unknown but it is associated with neurons lying within the fusiform cell layer. Here we investigated changes of excitability within identified fusiform cells following AOE. Wistar rats were exposed to a loud (110 dB SPL) single tone (14.8 kHz) for 4 h. Auditory brainstem response recordings performed 3–4 days after AOE showed that the hearing thresholds were significantly elevated by about 20–30 dB SPL for frequencies above 15 kHz. Control fusiform cells fired with a regular firing pattern as assessed by the coefficient of variation of the inter-spike interval distribution of 0.19 ± 0.11 (*n* = 5). Three to four days after AOE, 40% of fusiform cells exhibited irregular bursting discharge patterns (coefficient of variation of the inter-spike interval distribution of 1.8 ± 0.6, *n* = 5; *p* < 0.05). Additionally the maximal firing following step current injections was reduced in these cells (from 83 ± 11 Hz, *n* = 5 in unexposed condition to 43 ± 6 Hz, *n* = 5 after AOE) and this was accompanied by an increased firing gain (from 0.09 ± 0.01 Hz/pA, *n* = 5 in unexposed condition to 0.56 ± 0.25 Hz/pA, *n* = 5 after AOE). Current and voltage clamp recordings suggest that the presence of bursts in fusiform cells is related to a down regulation of high voltage activated potassium currents.

In conclusion we showed that AOE triggers deafness at early stages and this is correlated with profound changes in the firing pattern and frequency of the DCN major output fusiform cells. The changes here described could represent the initial network imbalance prior to the emergence of tinnitus.

## Highlights

► Wistar rats were exposed to 15 kHz, 110 dB SPL (acoustic over exposure, AOE). ► AOE triggered hearing loss in rats for frequencies exceeding 15 kHz. ► AOE triggered bursts in dorsal cochlear nucleus fusiform cells. ► AOE reduced high voltage activated K^+^ currents in those cells.

## 1 Introduction

Hearing loss triggered by acoustic over-exposure (AOE) is highly prevalent in our society and can affect quality of life through the emergence of phantom sounds or tinnitus (Loeb and Smith, 1967; Roberts et al., 2010). One of the major consequences of AOE is an increase of spontaneous neuronal activity observed at several levels of the mammalian auditory system, including the cochlear nucleus, the inferior colliculus and the auditory cortex (Zhang and Kaltenbach, 1998; Komiya and Eggermont, 2000; Brozoski et al., 2002; Norena and Eggermont, 2003; Seki and Eggermont, 2003; Ma et al., 2006).

Tinnitus in humans often persists following section of the auditory nerve (Barrs et al., 1984) suggesting that it could be of central origin. The cochlear nucleus is the first relay in the central auditory pathway and processes acoustic information from direct projections of the auditory nerve. Elevated activity in the dorsal part of the cochlear nucleus (DCN) is of interest because hyperactivity in this structure has been associated with the perception of tinnitus (Brozoski et al., 2002). Moreover hyperactivity in the DCN following AOE is maintained after removal of the auditory nerve (Zacharek et al., 2002). Hyperactivity in the DCN could either drive hyperactivity in the inferior colliculus and auditory cortex directly and/or trigger changes in synaptic function and intrinsic neuronal excitability that then generate hyperactivity in the higher parts of the auditory pathway.

Identification of the cell type(s) giving rise to hyperactivity in the DCN is therefore a critical step towards unravelling the cellular mechanisms underlying the development of tinnitus. A possible clue to the origin of hyperactivity in the DCN emerged from multi-unit recordings performed *in vivo*, which found that hyperactivity in the DCN induced by intense sound exposure was greater in the fusiform layer compared to other DCN layers (Brozoski et al., 2002; Kaltenbach and Falzarano, 2002). However although extracellular, multi-unit recordings can reveal the pattern of neuronal discharge (regular versus bursting), they are limited in their ability to reveal the neurone subtypes involved or the mechanisms that give rise to the observed hyperactivity. Indeed, the reported hyperactivity could reflect increases in single cell spontaneous discharge rates, but could also arise from an increase in the number of active neurons, changes in average spike amplitude or the emergence of burst discharges. Finlayson and Kaltenbach (2009) recently reported an increase in the incidence of simple spiking as well as increase in the incidence of spontaneous bursting in the DCN after AOE. In the DCN, principal fusiform cells (FCs) exhibit a regular pattern of firing whereas cartwheel cells uniquely exhibit a bursting pattern (Manis et al., 1994). Therefore the increase of simple spiking activity and the increase of bursting activity observed by Finlayson and Kaltenbach (2009) could be due to hyperactivity of FCs and cartwheel cells, respectively. An alternative possibility could be that hyperactivity originates from simple spiking FCs and that a proportion of FCs changes their firing pattern from regular to bursting. Consequently, we set out to use whole cell recordings from identified FCs in DCN slices in order to determine changes of excitability three to four days after AOE and characterize mechanisms behind those excitability changes.

## 2 Material and methods

### 2.1 Animals

Seventeen Wistar rats aged between 15 and 22 days old were used. Experiments were carried out in accordance with the UK Animals (Scientific Procedures) Act 1986 and the Home Office regulations.

### 2.2 Measurement of auditory brainstem response (ABR)

Rats were anesthetized with an intraperitoneal injection of fentanyl (0.15 mg/kg), fluanisone (5 mg/kg, VetaPharma Ltd) and Hypnovel (2.5 mg/kg, Roche). Positive, negative, and ground electrodes were inserted subcutaneously at the vertex, mastoid, and back, respectively (Murashita et al., 2006). Auditory brainstem responses were evoked by calibrated tone pips (8/12/16/24/30 kHz; 1 ms rise and fall times, 5 ms duration, 3 ms plateau) generated in a free field at 10 Hz by a waveform generator (TGA 1230 30 MHz, Tucker Davis Technology, USA) and an acoustic driver (Bruel & Kjaer type 4192, Denmark). Evoked responses were recorded by an amplifier (Medelec Sapphire 2A, Oxford Instruments, UK), band-pass filtered between 10 Hz and 5 kHz and averaged from 300 to 400 Hz sweeps or 800 to 1000 sweeps at threshold using custom made software (CAP, GSK). Tone pips were progressively attenuated in 10 or 3 dB SPL steps from an initial intensity of 94 dB SPL using a digital attenuator (PA4, Tucker Davis Technology, USA). Hearing threshold was defined as the lowest intensity yielding the consistent appearance of ABR peaks I and II. Thresholds shift used as the primary indicator of hearing performance was measured at the left ear as the difference between the hearing threshold on day 1 (P15-18) and the hearing threshold 3 or 4 days after the AOE procedure.

### 2.3 Acoustic over exposure (AOE)

Rats were anesthetized, as detailed above, and placed in a custom made open field sound-insulated chamber containing a 600 W High Power Horn Tweeter radiating evenly, freq range 2–20 kHz (Maplin UK*)* so that both ears were exposed. A pure tone of 14.8 kHz was delivered at 110 dB SPL for a total of 4 h. Two sessions of 2 h of AOE were performed at P15-P18 which corresponds to the period after the hearing onset (Geal-Dor et al., 1993) with a one day interval between the two sessions. Control animals were similarly anesthetized but unexposed to AOE.

### 2.4 Whole cell recordings

Whole cell recordings were here conducted at 3–4 days after the AOE (i.e. P18-22) as reliable recordings could only be obtained from juvenile rats. Recordings were performed within slices originating from two littermates on the same day (one control animal and one animal previously exposed to sound). The two littermates were tested for their hearing threshold before the *in vitro* recordings.

Coronal brainstem slices (250 μm) containing the DCN were obtained from Wistar rats (P18-22) and placed in low Na^+^ ACSF with 0.1 mM Ca^2+^ and 4 mM Mg^2+^, as previously described (Barnes-Davies et al., 2004). Current and voltage clamp whole cell recordings were obtained from FCs and cartwheel cells identified on the basis of their morphological and electrophysiological properties (Oertel and Wu, 1989; Pilati et al., 2008). Whole cell recordings were performed using a Multiclamp 700 A amplifier (Molecular Devices Inc. USA), with a sampling rate of 20 kHz, filtered at 5 kHz, and using PClamp 9 software (Molecular Devices Inc. USA). When studying the effects of AOE, only cells found in the high-frequency region of the DCN were selected (Yajima and Hayashi, 1989).

Current clamp recordings were carried out in normal ACSF (Barnes-Davies et al., 2004) with 2 mM Ca^2+^ and 1 mM Mg^2+^. Voltage clamp recordings were carried out in ACSF containing 0.5 mM CaCl_2_, 2.5 mM MgCl_2_ and 0.5 μM tetrodotoxin to study Kv K^+^ currents in isolation from K_Ca_ and Na^+^ currents. The pipette (4–6 MΩ) contained (in mM): Kgluconate 97.5; KCl 32.5; EGTA 5.4; HEPES 10; MgCl_2_ 1; NaCl 2; 0.1% Lucifer yellow (adjusted to pH of 7.1–7.3 with KOH). Signals were corrected off-line for the liquid junction potential (−11 mV). Series resistance <12 MΩ was compensated by 70%. All recordings were performed at 25 °C. High voltage activated K^+^ currents were elicited by applying step commands (from −70 mV to +30 mV in 10-mV increments) from a pre-pulse voltage (−30 mV, 1 s) (Brew and Forsythe, 1995).

### 2.5 Spike analysis

Coefficient of variation of inter-spike intervals (ISI) relative to the spontaneous rate of firing was calculated as the ratio of the standard deviation to the mean of the ISI. Firing rates after step current injections were fitted with a sigmoidal function $y=a/\left(1+\exp^{\left(-\left(x-x_{0}\right)/b\right)}\right),$ where *x* is the current (in pA), *x*_0_ is the point of inflection of the curve, *y* is the frequency (in Hz), *a* is the maximal frequency and *b* is the slope (firing gain). Firing rates after synaptic stimulations (Input–output relationships) were fitted by a Hill equation $y=F_{\max}/\left(1\;+\;10^{\left(\left(\text{Log}\;X_{50}-X\right)*n\right)}\right),$ where *y* is the response (Hz), *F*_max_ is the maximum firing rate, *X* is the logarithm of the input frequency (Hz), *X*_50_ is the values of *X* at which F reaches half maximum, and *n* is the Hill coefficient (slope).

### 2.6 Statistical analysis

One-way ANOVA tests were used to test for differences in the action potential firing properties (Tables 1 and 2) among three populations. This was followed by a Tukey post Hoc test to assess the degree of significance between the populations. Comparison between voltage clamp K^+^ currents obtained in control and in AOE was made with the Student *t*-test for unequal variances. Paired *t*-tests were used to compare K^+^ currents before and after exposure to TEA. The level of significance was set at 0.05.

## 3 Results

### 3.1 Fusiform cells in the DCN fire simple and regular action potentials

Whole cell current clamp recordings were obtained from fusiform cells (FCs) within the DCN. Under control conditions (rats without AOE), FCs fired spontaneous action potentials (Fig. 2A and B) with a threshold of around −65 mV (Table 1). Action potentials were characteristically followed by an undershoot, similar to that observed previously (Hirsch and Oertel, 1988; Zhang and Oertel, 1994). More depolarised holding potentials were associated with higher frequency firing (23 ± 5 Hz, *n* = 5 cells, 3 rats) and a decreased coefficient of variation (CV) of the inter-spike interval (ISI) (Fig. 2B, Table 1). The ISI distribution was consistently monomodal for all holding potentials and firing frequencies (Fig. 2A and B).

### 3.2 After AOE a subset of fusiform cells fire complex and irregular action potentials (bursts)

**Fig. 1:**
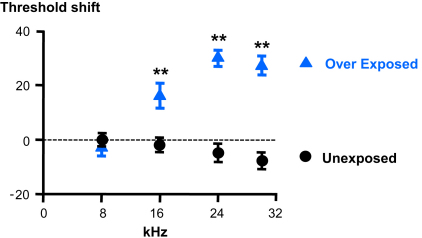
Exposure to a 14.8 kHz tone (110 dB SPL) increases the hearing threshold for frequencies exceeding 8 kHz. Wistar rats (P15-17) were either exposed at day 0 or similarly anesthetized but unexposed to the single tone. Summary of auditory brainstem response thresholds shifts (see Methods) for 8–30 kHz frequencies from 13 unexposed and 20 exposed rats. **P < 0.01, unpaired *t* student test.

**Fig. 2:**
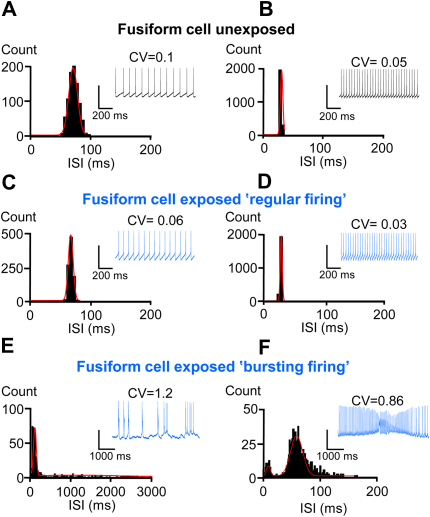
Acoustic over-exposure triggers an irregular spontaneous firing pattern in a proportion of DCN fusiform cells (FCs). Histograms show the inter-spike interval (ISI) distributions (from unexposed and exposed rats) with corresponding traces in inset as FCs are firing at threshold (A, C, E) and at their maximal firing frequency (B, D, F) when depolarized. Above each trace the coefficient of variation (CV) relative to the distribution is shown. A, B. A regular firing pattern is observed in a FC from an unexposed rat with ISI distributions fitted with a Gaussian function. C, D. Example of a FC firing with a regular pattern after AOE with ISI distributions fitted with a Gaussian function. E, F. Example of a FC firing with bursts after AOE with ISI distributions fitted with a double Gaussian function. Similar pattern was observed in 6/22 FCs after AOE. Holding potentials were −62 mV (A), −55 mV (B), −61 mV (C), −56 mV (D), −68 mV (E), −58 mV (F); maximal firing frequencies were 30 Hz (B), 32 Hz (D), 20 Hz (F); ISI at the peak were 86 ms (A), 33 ms (B), 65 ms (C), 33 ms (D), 34 and 1100 ms (E), 10 and 90 ms (F). Vertical calibration bars: 40 mV.

**Fig. 3:**
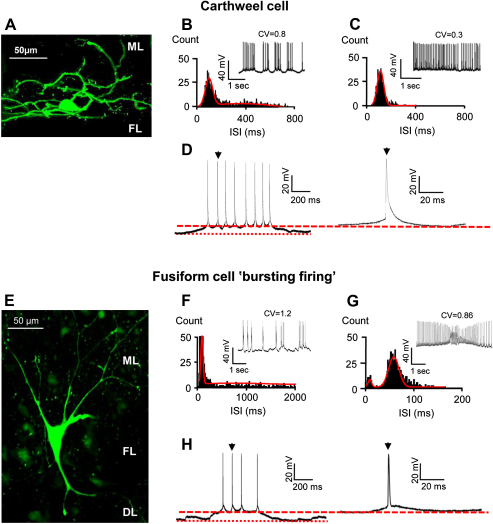
Example of a cartwheel cell (A–D) and a fusiform cell (E–H) firing with bursts after AOE. A. Cartwheel cell filled with lucifer yellow lying between the fusiform layer (FL) and the molecular layer (ML) characterised by its large spiny dendritic tree. B–D. Cartwheel cell firing with bursts in control conditions with ISI distributions fitted with a double (B) or a single (C) Gaussian function. Example of a burst of action potentials recorded in this cartwheel cell and a single action potential within the burst (D, arrowhead). E. Fusiform cell filled with lucifer yellow lying in the FL characterised by its fusiform shape. Its basal dendrites project towards the deep layer (DL) while the apical dendrites are orientated towards the ML. F–H. Fusiform cell firing with bursts after acoustic over-exposure with ISI distributions fitted with a double Gaussian function (F, G). Example of a burst of action potentials recorded in this fusiform cell and a single action potential within the burst (H, arrowhead). The dashed lines in D and H represent the baseline from which measurements for the action potential were taken whereas the dotted lines represent the baseline of the burst. Membrane potentials were −67 mV (B), −63 mV (C), −68 mV (F), −58 mV (G); maximal firing frequencies were 24 Hz (C), 20 Hz (G); ISI at the peaks were 98 and 400 ms (B) 117 ms (C) 34 and 1100 ms (F), 10 and 90 ms (G). Above each trace the coefficient of variation (CV) relative to the distribution is shown.

Previous studies reported increased spontaneous activity within the DCN following AOE (Zhang and Kaltenbach, 1998; Kaltenbach and Falzarano, 2002). However whether this involves changes in local interneuron activity or FC intrinsic excitability is unknown. Five Wistar rats subjected to AOE of 110 dB SPL at 14.8 kHz for 4 h showed a shift of their hearing threshold for frequencies above 15 kHz, 3–4 days after the AOE (Fig. 1). After AOE, 60% of FCs exhibited a spontaneous regular firing pattern and maximal firing frequencies of 29 ± 3 Hz (*n* = 7 out of 12 cells, 5 rats) similar to FCs from unexposed rats (Fig. 2C and D and Table 1). However, for the remaining 40% FCs, spontaneous firing was characterised by bursts of action potentials generally occurring on top of small (12 ± 1.2 mV, *n* = 5 out 12 cells, 5 rats) transient depolarizations (Fig. 2E and F). Bursting FCs were characterised by the absence of an AP undershoot following the action potentials and a maximal firing frequency of 15 ± 2 Hz (*n* = 5 cells) (Table 1). The ISI plots characterising bursting FCs after AOE showed a bimodal distribution (Fig. 2E and F) at all holding potentials, and a 2-fold larger CV (1.8 ± 0.6, *n* = 5 cells) compared to regular firing FCs from exposed (0.05 ± 0.01, *n* = 7 cells, *P* < 0.05) and unexposed rats (0.19 ± 0.1, *n* = 5 cells, Table 1, *P* < 0.05). The passive properties of the bursting FCs were unaffected compared to regularly firing FCs (Table 1), but action potentials were of smaller amplitude and longer (90-10%) decay time in comparison to FCs that displayed regular firing (Table 1, *P* < 0.05). To exclude the possibility that bursting FCs were actually cartwheel cells (Manis et al., 1994; Oertel and Young, 2004), we analyzed the pattern and firing property of 8 cartwheel cells (Fig. 3 and Table 1). Cartwheel cells and FCs were identified by their location in the slice as well as by their morphology assessed by Lucifer yellow filling (examples in Fig. 3A and E). When maintained at threshold, cartwheel cells displayed a bimodal ISI distribution (Fig. 3B) similar to that observed in bursting FCs (Fig. 3F). However when further depolarised, the distribution of the ISI became monomodal (with corresponding smaller CV, Fig. 3C, *P* < 0.05) in contrast to bursting FCs that maintained a bimodal distribution independent of the holding potential (Fig. 3G). Finally, cartwheel cells were also characterized by longer action potential decay times compared to bursting FCs (Fig. 3D and H, Table 1, *P* < 0.05).

### 3.3 Acoustic over-exposure leads to modulation of FC transfer function

**Fig. 4:**
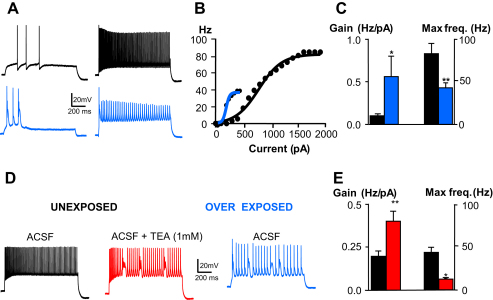
Effects of AOE on FC transfer function are reproduced by blocking high voltage activated (HVA) K^+^ currents. A–C. AOE decreases the maximal firing frequency in FCs. Traces in A represent the FC firing at maximal frequency in response to 1 s step current injection in unexposed (black) and over-exposed condition (blue). (B) FC transfer function for the same cells shown in A, B. Gains were 0.05 Hz/pA (unexposed) and 0.2 Hz/pA (exposed), maximal frequencies were 84 Hz (unexposed), 40 Hz (exposed). C. Summary histograms representing the firing gain and the maximal firing frequencies for unexposed (*n* = 5, black) and exposed conditions (*n* = 5, blue). **P < 0.01, *P < 0.05 (unpaired *t* test). D. TEA (1 mM) triggers bursts in FCs similarly to AOE. E. Summary histograms representing the firing gain and the maximal firing frequencies for FCs recorded in ACSF (black) and following application of TEA (red) (*n* = 3, *P < 0.05, **P < 0.01 paired *T* tests). Membrane potentials were −80 mV. (For interpretation of the references to colour in this figure legend, the reader is referred to the web version of this article.)

The way a neuron processes signals can be captured by its transfer function, or input–output relationship (Koch, 1999). Modulation of excitability changes the shape of this relationship which can affect either the slope (gain) or the output maximum (Holt and Koch, 1997; Chance et al., 2002). We investigated the transfer function for FCs by examining their firing frequency in response to depolarizing current pulses. FCs from unexposed rats fired trains of action potentials in response to step current injections, reaching a maximal firing frequency of 83 ± 11 Hz (*n* = 5 cells) with a gain of 0.09 ± 0.01 Hz/pA (*n* = 5) (Fig. 4A, Table 2), consistent with values previously observed in DCN FCs (Manis, 1990; Zhang and Oertel, 1994). In contrast after AOE, bursting FCs reached a maximal firing frequency of just 43 ± 6 Hz (*n* = 5 cells, *P* < 0.01 compared to cells from unexposed rats) and with a gain of 0.56 ± 0.25 Hz/pA (*n* = 5 cells, *P* < 0.05, Fig. 4A–C and Table 2). No differences were observed between FCs from unexposed rats and the non-bursting FCs from over-exposed rats (Table 2).

### 3.4 AOE down-regulates high voltage activated potassium currents

**Fig. 5:**
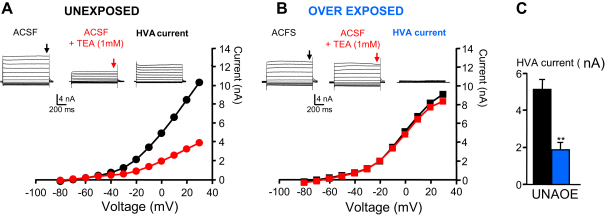
HVA K^+^ currents are down regulated after AOE. Representative current traces and current–voltage relationship for HVA K^+^ currents in unexposed (A) and exposed (B) conditions in absence (ACSF) and in presence of TEA (ACSF + TEA 1 mM). The HVA K^+^ currents were obtained by subtracting the current recorded in the presence of TEA from the current measured in ACSF C. Summary histograms representing the HVA K^+^ current measured at +30 mV in unexposed (black) and exposed (blue) condition **P < 0.01, unpaired *t* test. (For interpretation of the references to colour in this figure legend, the reader is referred to the web version of this article.)

Since high voltage activated (HVA) K^+^ currents have been shown to permit high-frequency firing of neurons (Brew and Forsythe, 1995; Wang et al., 1998; Rudy and McBain, 2001; Lien and Jonas, 2003), we used 1 mM TEA to selectively inhibit these currents in FCs from unexposed rats. Under these conditions, depolarizing current pulses evoked bursts of action potentials (Fig. 4D) with a reduction in the maximal firing frequency and an increase in the firing gain similar to that observed in bursting FCs after AOE (Fig. 4E, *P* < 0.01). Block of HVA K^+^ currents with TEA produced bursts in otherwise regularly firing FCs, suggesting that bursts after AOE are due to down regulation of HVA K^+^ currents. We used voltage clamp to quantify HVA K^+^ currents from FCs from unexposed and over-exposed rats. Voltage steps more positive than −30 mV from a holding potential of −70 mV evoked HVA K^+^ currents that were sensitive to 1 mM TEA in both control and AOE conditions (Fig. 5A and B). However, the TEA-sensitive component of the HVA K^+^ current of FCs was significantly smaller after AOE (1.9 ± 0.4 nA, *n* = 9 cells, 3 rats) compared to FCs from unexposed rats (5.1 ± 0.5 nA, *n* = 6 cells, 3 rats *P* < 0.001 Student’s *t* test, Fig. 5C). This supports the idea that AOE causes down regulation of HVA K^+^ currents that are likely responsible for the presence of bursts.

## 4 Discussion

An increase in neuronal activity concomitant to tinnitus induced by AOE is reported in the DCN (Kaltenbach et al., 1998; Zhang and Kaltenbach, 1998) but the cellular origin remains unknown. Our findings now show that the intrinsic electrical properties of FCs in the DCN are modified following exposure to loud sound. After AOE, a proportion of FCs exhibit a distinct bursting firing pattern, and thereby lose the ability to fire regularly or at high firing frequencies. Our results suggest that one mechanism contributing to this change of activity is the down regulation of HVA K^+^ currents in FCs.

### 4.1 Regular firing pattern of fusiform cells

The observations made with here with whole cell recordings were related to the intrinsic properties of DCN FCs in response to constant or step current injections (i.e. independently of their synaptic connections). FCs were capable of firing reliable and precise trains of action potentials in response to depolarizations confirming results obtained in previous *in vitro* studies (Manis, 1990; Oertel and Wu, 1989) and also *in vivo* studies (Ding et al., 1999; Hancock and Voigt, 2002). Studies of Shore et al. (2008) in guinea pigs and by Brozoski et al. (2002) in chinchillas, showed that units displaying increased spontaneous discharge rates after intense noise exposure, exhibited pauser/buildup patterns that are generally considered to be typical of fusiform cells. The lack of any increase in the spontaneous firing rates of individual neurons of intense-tone-exposed rats compared to those of control rats reported here therefore contrasts with the results from *in vivo* experimentation. This could be due to two factors. First, the firing patterns of FCs *in vivo* can reflect synaptic events occurring in the complex DCN network via its various afferent projections (Oertel and Young, 2004; Shore et al., 2008). There is a greater amount of synaptic noise *in vivo* that contributes to the spontaneous activity in afferent neurons by either increasing or decreasing the variability of inter-spike intervals. For example, low afferent rates with large synaptic events generate an increase in the variability of the inter-spike interval distribution (Calvin and Stevens, 1968).

Second, the shape of the synaptic current injection produced by acoustic stimuli could respond with a higher probability of discharge in response to a tone onset, corresponding to the high firing rate at the onset transient of auditory nerve fibres (Kiang et al., 1965; Smith and Brachman, 1980). Therefore the spontaneous discharge rates of regularly firing fusiform cells could stay unaffected after AOE using *in vitro* recordings due to the limited synaptic connections and to the shape of the current injection.

### 4.2 Presence of bursts in fusiform cells after acoustic over-exposure

A previous study reported an increase bursting activity in slices from exposed rats (Chang et al., 2002). As recordings were performed extracellularly, cell types involved in that bursting activity could not be identified. Since regular neurons include fusiform cells (Manis, 1990; Zhang and Oertel, 1994) and bursting neurons include cartwheel cells (Manis et al., 1994) it was hypothetised that intense tone exposure leads to increased activity of DCN cartwheel cells (Chang et al., 2002). The authors also suggested that activity of some fusiform cells might change to bursting and considered that this was unlikely as regular firing of DCN neurons never changed to bursting despite a large variety of manipulations (Waller et al., 1996; Chen et al., 1998, 1999). Our study shows that after AOE, a proportion of identified FCs loses the ability to fire regularly and fire irregular bursts instead. We cannot exclude that bursting cartwheel cells were not increased after AOE as suggested by Chang et al. (2002) since we did not record from those cells after AOE. Moreover an increase of spontaneous activation of cartwheel cells would lead to a reduction of the spontaneous discharge rates of regularly firing fusiform cells (Chang et al., 2002) which was not observed here.

Finlayson and Kaltenbach (2009) examined the effects of intense single tone exposure on multi-unit spontaneous activity in the DCN. Recordings conducted 5–6 days after the exposure revealed a 30% increase in the incidence of bursts. The cellular origin of those bursts remained unknown and it was hypothesised that bursts could be due to an increased activity of FCs. This proportion is consistent with our finding as bursts were observed only in 40% FCs 3–4 days after AOE.

Juvenile rats were here used and hearing loss at an early age can cause a much more severe impairment on neural plasticity (Sanes and Kotak, 2011) audiogenic seizures (Pierson and Liebmann, 1992) and acoustic startle reflex (Rybalko et al., 2011). However the presence of bursts has also been reported in adult animals (Finlayson and Kaltenbach, 2009) and it is therefore possible that the presence of bursts reflect the severity of the acoustic trauma in juvenile or in adult animals.

Each FC is excited by a small group of tonotopically organized auditory nerve fibres (Yajima and Hayashi, 1989). Thus we can speculate that the subpopulation of FCs found to fire in bursts after AOE are those that process information from auditory nerve fibres associated with frequencies above 15 kHz (the frequency used during the AOE). Although all FCs recorded in this study were localized in the 15–50 kHz region (methods), an accurate estimation of the precise location within the 15 kHz band would be possible only with *in vivo* recordings where responses to input tones of different frequencies could be monitored. However, aside from a tonotopic explanation from the small percentage of affected FCs post-AOE, it is also possible that the switch of firing pattern is a gradual process that continues beyond the three to four days post-AOE interval over which the presence study was conducted.

### 4.3 Mechanisms underlying bursts

Our study demonstrates that intense sound stimulation could induce a significant reduction in TEA-sensitive HVA K^+^ currents in DCN FCs. This change in the intrinsic properties of FCs could contribute to the emergence of a burst firing pattern in those cells. Indeed, bursts could be reproduced in FCs from control (unexposed) rats by adding 1 mM TEA to the superfusion medium to block HVA K^+^ channels (Rudy and McBain, 2001; Johnston et al., 2010). High voltage activated K^+^ currents enable cells to fire at high rates (Rudy and McBain, 2001). We found that the addition of TEA reduced the frequency of the action potential frequency in DCN FCs and a similar effect was also observed in DCN FCs after AOE. By activating at depolarized potentials and rapidly deactivating, HVA K^+^ currents facilitate action potential repolarisation and lead to a short action potential duration. We found that FCs with a bursting pattern of firing exhibited significantly longer action potential decay times compared to unexposed FCs, which is consistent with a down regulation of HVA K^+^ currents. DCN neurons express high levels of mRNA for the Shaw-related potassium channel subunits Kv3 (Friedland et al., 2007). As these subunits exhibit high activation voltage properties and are TEA-sensitive (Grissmer et al., 1994; Brew and Forsythe, 1995; Leão et al., 2010) this suggests that the HVA K^+^ currents described here are carried by those Kv3 K^+^ subunits. TEA-sensitive Kv1 Shaker-related K^+^ subunits characterized by low activation voltage are also expressed in the DCN (Grissmer et al., 1994; Hopkins, 1998). However in our experimental conditions those currents were inactivated by the depolarized pre pulses at −30 mV (Brew and Forsythe, 1995). We can also exclude slow delayed rectifier (Kv2) K^+^ currents that are activated at high voltages but are insensitive to 1 mM TEA (Johnston et al., 2010) and do not appear to be expressed in the DCN (Friedland et al., 2007). Kv3 K^+^ channels have been previously shown to be modulated by activity in the auditory brainstem (Steinert et al., 2011). In the medial nucleus of the trapezoid body, short *in vivo* sound stimulations increase the functional expression of Kv3.1b K^+^ channel subunits thereby altering the firing properties of those neurons (Leão et al., 2010). Changes in the acoustic environment also affect the phosphorylation state of Kv3.1b K^+^ channel subunits which can increase and decrease within minutes (Song et al., 2005). Moreover studies in congenitally deaf mice showed that the normal tonotopic distribution of Kv3.1 K^+^ channels was absent in the medial nucleus of the trapezoid body (von Hehn et al., 2004; Leao et al., 2006). It is therefore likely that the modulation of Kv3 K^+^ currents represents a mechanism adjusting neuronal intrinsic excitability to the synaptic excitability. In epilepsy the absence of the Kv3 subunit prevents interneurons from exhibiting normal rapid firing patterns and causes disinhibition of target neurons (Lau et al., 2000). A similar mechanism could occur in the DCN and could lead to the pathological changes observed in the present study which may account for the abnormal cell activity which has been associated with tinnitus (Brozoski et al., 2002; Kaltenbach, 2007; Kaltenbach and Godfrey, 2008). However, most *in vivo* studies relating behavioural evidence of tinnitus with hyperactivity in the DCN were performed in adult animals (Brozoski et al., 2002; Middleton et al., 2011). Moreover, tinnitus was generally induced after exposing one ear to loud sound and loud sound applied to the two ears may here limit the onset of tinnitus. Finally, the increase of cellular excitability within the DCN was recorded several weeks after the noise exposure (Brozoski et al., 2002; Middleton et al., 2011). Although Finlayson and Kaltenbach (2009) recorded bursts 5–6 days after exposing both ears to loud sound, a direct link between bursts and the hyperactivity in the DCN related to tinnitus remains to be established.

### 4.4 Bursts induced by acoustic over exposure, a result of homeostatic plastic adjustments within the DCN?

Homeostatic plasticity stabilizes the properties of neuronal circuits by regulating neuronal excitability (Turrigiano, 1999). In the hippocampus, the competition between single spike firing and burst firing represents a homeostatic regulatory mechanism to maintain synaptic strength and consequently the firing rate in pyramidal cells (Buzsaki et al., 2002). A similar switch between tonic and bursting firing has also been shown in invertebrate preparations (Turrigiano et al., 1995). Therefore hyperactivity in the DCN and/or bursts could be the result of homeostatic plastic adjustments to restore the stability of the DCN network following a reduced auditory nerve activity after acoustic over-exposure (Dallos and Harris, 1978; Salvi et al., 1983; Schaette and Kempter, 2009). As the regular pattern of activity could compete with the presence of bursts neurons to maintain the physiological condition, this would explain why bursts affect only a proportion of fusiform cells within the DCN. In conclusion acoustic over exposure would trigger harmful bursting within DCN fusiform cells leading to tinnitus similarly to traumatic brain injuries triggering abnormal hyperexcitability leading to acute seizures and epilepsy (Topolnik et al., 2003; Timofeev et al., 2010).

### 4.5 Kv3, a target against tinnitus?

So far the neuronal mechanisms of tinnitus are not fully understood and therefore there are no effective treatments available. Several approaches have been used to reduce the tinnitus symptoms such as local application of the local anaesthetic lidocaine (Trellakis et al., 2007), administration of benzodiazepines and GABAergics agonists (Witsell et al., 2007), Ca^2+^ channel antagonists (Theopold, 1985; Davies et al., 1994) and NMDA receptor antagonists (Ehrenberger, 2005). Multiple hypotheses have also been raised concerning the central or peripheral mechanisms that are responsible for tinnitus after an acoustic insult (Nicolas-Puel et al., 2002; Eggermont and Roberts, 2004; Kaltenbach and Godfrey, 2008). It is likely that tinnitus starts at the peripheral level and evolves throughout the central auditory pathway via a process that resembles memory consolidation. If this is the case, treating the first symptoms linked to acoustic over-exposure could be proven effective in slowing developing tinnitus at a later stage. The data provided here suggest that the Kv3 K^+^ channels in the central auditory system could be a target responsible for tinnitus at the early stages following acoustic over-exposure.

## Figures and Tables

**Table 1:** Active and passive properties of fusiform (FC) and cartwheel cells (CW) in unexposed conditions and FC after AOE (regular firing and bursting). Action potential firing properties were quantified by the frequency, the coefficient of variation (CV) and the membrane potential (*V*_m_) measured at action potential threshold, half maximal frequency (½ *F*_max_) and maximal frequency (*F*_max_). Action potential characteristics were described by the amplitude, 10%–90% rise time and 90%–10% decay time. Passive properties were the resting potential, the membrane resistance and the capacitance. The number of cells showing an undershoot in the repolarising phase has been reported in the last row. Column “FC AOE regular”: value under bracket refers to comparison with values in the column “FC unexposed”. Column “FC AOE bursting”: first and second values under bracket refer to comparison with values in the column “FC unexposed” and “FC AOE regular” respectively. Column “CW unexposed”: value under bracket refers to comparison with values in the column “FC AOE bursting”. *P* values were obtained with ANOVA one-way (Tukey’s post-hoc) tests. N.S. = non significant for *P* values > 0.05.

	FC unexposed (*n* = 5)	FC AOE ‘regular’ (*n* = 7)	FC AOE ‘bursting’ (*n* = 5)	CW unexposed (*n* = 8)
Firing frequency (Hz) at threshold	0.2 ± 0.1	0.5 ± 0.1 (NS)	3.4 ± 1.9 (NS, NS)	0.8 ± 1.3 (NS)
Coefficient of variation at threshold	1.7 ± 0.1	1.9 ± 0.4 (NS)	4.4 ± 1.4 (NS, NS)	1.6 ± 0.3 (*P* < 0.05)
*V*_m_ (mV) at threshold	−65.0 ± 1.8	−61.0 ± 1.9 (NS)	−74.0 ± 2.3 (*P* < 0.05, *P* < 0.01)	−72 ± 2.0 (NS)
Firing frequency (Hz) at ½ *F*_max_	9.0 ± 1.7	11.0 ± 2.0 (NS)	5.0 ± 2.7 (NS, NS)	
CV at ½ *F*_max_	0.19 ± 0.04	0.15 ± 0.40 (NS)	3.05 ± 1.90 (*P* < 0.01, *P* < 0.01)	
*V*_m_ (mV) at ½ *F*_max_	−50.0 ± 2.2	−48.0 ± 1.6 (NS)	−50.0 ± 1.4 (NS, NS)	
Firing frequency (Hz) at *F*_max_	23.0 ± 4.7	29.0 ± 2.5 (NS)	15.0 ± 2.1 (NS, NS)	15 ± 1.5 (NS)
CV at *F*_max_	0.19 ± 0.1	0.05 ± 0.01 (NS)	1.80 ± 0.6 (*P* < 0.05, *P* < 0.01)	0.5 ± 0.1 (*P* < 0.05)
*V*_m_ (mV) at *F*_max_	−44.0 ± 2.9	−43.0 ± 1.9 (NS)	−53.0 ± 3.1 (NS, NS)	−64 ± 2.0 (NS)
Amplitude (mV)	91.0 ± 7.7	88.0 ± 3.7 (NS)	69.0 ± 2.5 (*P* < 0.05, *P* < 0.05)	60 ± 4.6 (NS)
10–90% rise time (ms)	1.3 ± 0.8	0.9 ± 0.3 (NS)	0.6 ± 0.1 (NS, NS)	0.6 ± 0.06 (NS)
90–10% decay time (ms)	0.8 ± 0.1	0.7 ± 0.1 (NS)	2.3 ± 0.1 (*P* < 0.001, *P* < 0.001)	7.3 ± 1.3 (*P* < 0.05)
Resting potential (mV)	−47.0 ± 2.5	−54.0 ± 3.9 (NS)	−49.0 ± 1.4 (NS, NS)	−51.0 ± 1.8 (NS)
Membrane resistance (mΩ)	120.0 ± 40.0	89.0 ± 28.0 (NS)	113.0 ± 23.0 (NS, NS)	128.0 ± 23.0 (NS)
Capacitance (pF)	151.0 ± 36.0	167.0 ± 26.0 (NS)	117.0 ± 10.0 (NS, NS)	89.6 ± 10.5 (NS)
Undershoot (number of cells)	5	5	0	0

**Table 2:** Action potential firing properties in response to step current injections in FCs in the unexposed condition and after AOE (regular and bursting). Following values are reported: minimal injected current to elicit an action potential (I threshold), voltage reached to elicit an action potential from a membrane potential of −80 mV (V threshold). Last row refers to the number of FCs displaying an undershoot. Column “FC AOE regular”: value under bracket refers to comparison with values in the column “FC unexposed”. Column “FC AOE bursting”: first and second values under bracket refer to comparison with values in the column “FC unexposed” and “FC AOE regular” respectively. *P* values were obtained with ANOVA one-way (Tukey’s post-hoc) tests. N.S. = non significant for *P* values >0.05.

	FC unexposed (*n* = 5–9)	FC AOE ‘regular’ (*n* = 5–7)	FC AOE ‘bursting’ (*n* = 5)
Maximal firing frequency (Hz)	83 ± 11	100 ± 23 (NS)	43 ± 6.0 (*P* < 0.05, *P* < 0.05.)
Gain (Hz/pA)	0.09 ± 0.01	0.06 ± 0.01 (NS)	0.56 ± 0.25 (*P* < 0.05, *P* < 0.05.)
I threshold (pA)	258 ± 43	264 ± 52 (NS)	83 ± 15 (*P* < 0.05, *P* < 0.05)
V threshold (mV)	−54 ± 2.0	−51 ± 1.0 (NS)	−53 ± 4.4 (N.S., N.S.)
Undershoot (number of cells)	9	7	0
